# A Good start in life: Effectiveness of integrated multicomponent multisector support on early child development—Study protocol

**DOI:** 10.1371/journal.pone.0267666

**Published:** 2022-08-03

**Authors:** Vicky Saunders, Maddison Beck, Jacqueline McKechnie, Michelle Lincoln, Christine Phillips, Jane Herbert, Rachel Davey

**Affiliations:** 1 Health Research Institute, University of Canberra, Bruce, ACT, Australia; 2 Faculty of Health, University of Canberra, Bruce, ACT, Australia; 3 Australian National University, Canberra, ACT, Australia; 4 University of Wollongong, Northfields Avenue, Wollongong, NSW, Australia; Xiamen University - Malaysia Campus: Xiamen University - Malaysia, MALAYSIA

## Abstract

**Introduction:**

Early childhood experiences have a lifelong impact on a child’s future. Social and environmental experiences and interactions have a profound relational effect on children’s physical and mental health which transfers agency to parents, caregivers and duty-bearers to care for the child’s welfare. In the Australian context early child development indices have been in decline in some communities. Hence, there is a sense of urgency to reverse these trends from an integrated perspective. A multisector, multi component program of interventions named *A Good Start in Life* is proposed and is being tested in the Australian Capital Territory across suburbs with high levels of early childhood development disadvantage. The aim of this study is to evaluate the outcomes and processes related to targeted interventions, designed to integrate child and family services within the local district and embed allied health programs into early childhood education, care services and playgroups.

**Methods and analysis:**

The *Good Start in Life* study will use a quasi-experimental design (with a matched control geographical area) consisting of a combination of interventions that will build multisectoral collaboration across education, health and social services that connect and support families with children from birth to 5 years. The control area will be matched on demographic characteristics and early child development outcomes and trends over the pre-intervention period. Evaluation data will be collected at baseline, and then on an annual basis for a further three years. A mixed methods approach will be used to evaluate delivery processes: quantitative (checklists, questionnaires) and qualitative methods (observations, focus groups and key stakeholder interviews). Effectiveness of the programme will be evaluated by comparing early child development outcomes between the comparator areas from the Australian Early Development Census in 2024. The primary focus will be on reducing the number of children who are developmentally vulnerable on at least one early development index (EDI). Separate tests will be conducted for significant differences in the percentage of children at risk in each of the five individual EDI domains. These domains are physical health and wellbeing, social competence, emotional maturity, language and cognitive skills, and communication and general knowledge.

**Trial registration:**

ACTRN12621001140842.

## Introduction

Early childhood experiences and interactions have a profound influence on a child’s physical, cognitive, social and emotional development [1, 2] and form the critical foundation for their lives [3–5]. The social circumstances into which children are born determines their exposure to events that are either beneficial or detrimental to their development [6]. How well children grow and thrive depends on the nurturing conditions of their environment in the widest sense, and this is fundamentally important in the early years (under 5 years) when the brain and body are developing rapidly [7]. The primary provider of beneficial early development opportunities are the child’s parents and caregivers. However, most parents cannot provide strong supportive environments without the assistance of formal and informal support services When parental support is compromised, for example due to factors such as sustained poverty, family break-up, family violence, abuse, neglect, illness or disability, then some of the child’s beneficial development opportunities must be provided by external support mechanisms. Unfortunately, contemporary trends indicate that the prevalence of such adverse circumstances in Australia have been worsening [8].

In response to this issue, the Australian government established the Australian Early Development Census (AEDC) in 2009, to provide a national measurement programme to monitor Australian children’s development. The AEDC is undertaken every three years. There have been four waves of measurement, the last one being completed in 2018. The census involves teachers of children in their first year of full-time school (age 5–6 years) completing the Australian version of the Early Development Instrument [9]. The Instrument consists of approximately 100 behavioural and competency-based questions and collects data relating to five key areas of early childhood development, referred to as ‘domains’. These include: physical health and well-being; social competence; emotional maturity; language and cognitive skills (school based); communication skills and general knowledge.

Over the last three censuses (2012–2018), the Australian Capital Territory (ACT) has seen an increase in the proportion of children who are developmentally vulnerable on all but one of the AEDC domains and the two summary domains, vulnerable on one or more domains and vulnerable on two or more domains [10]. As a result, early childhood development has been a focus recently in the ACT. Early investigations have shown that services, organisations, and sectors continue to operate in isolation, and, as a result, it has been reported that families struggle to access cohesive and holistic pathways of care for their children. Examining the variation in early childhood vulnerability across suburbs making up the ACT’s nine districts reveals greater developmental vulnerability is experienced in clusters of suburbs. According to the 2018 AEDC, 26.4% of 5-year-old children in the Belconnen district were developmentally vulnerable in one or more domains [11].

An inventory of all early childhood services in the Belconnen district in 2019 revealed that there were multiple services across the education, health, and social services sectors in operation. These included but were not limited to Early Childhood Education and Care (ECEC) services, playgroups, preschools and parenting programs. However, evidence suggests that vulnerable families are unable to or choose not to access these services, or are not aware of them [12]. The fragmented nature of service delivery and accessibility, particularly across centres and services, hinders referrals and restricts pathways of care for children with developmental needs. Under these conditions developmental issues are not addressed, while the number of children entering school with significant social, communication, behaviour and health challenges continues to increase. Research confirmed that when such challenges are present on entering formal education, there are cascading effects throughout the school years and later in life [3, 4]. These children are more likely to experience poor academic achievement, early drop-out from school and unemployment [13].

Literature on integration between early childhood centres reports that improving collaboration between services by creating more connected and productive early childhood programmes and networks provides better opportunities for positive outcomes for children and families [14]. Further studies have shown that the creation of a “virtual” network in which services do not need to relocate to a singular location produces the same benefits as co-located services [15].

As the nature of service integration prohibits a singular definition, a fit-for-purpose, context-and cultural- specific definition needs to be developed. In this project, the development of a definition will be considered as an outcome as this is an iterative process and will be refined throughout the project to represent the context, nature, and function of the integrated network most accurately. Broadly, this project views service integration as the creation of or strengthening of agency between early childhood services and programs across sectors within the local area. Such integration should provide a more comprehensive connection for families and greater numbers and quality of developmental opportunities for children, leading to better outcomes.

This project aims to evaluate an intervention designed to integrate child and family services within the district and embed allied health programs into early childhood education and care services and playgroups and to evaluate the outcomes and processes of the intervention. The Project Management Team (PMT) collaborate with local services to form a network of related services for children aged between birth and 5 years across the health, education, and social services sectors, allowing families to more easily access and navigate through different services.

The two main aims for this project are:

1. To create an integrated network of services for young children and families in target suburbs in the Belconnen district in the ACT.
2. To reduce the proportion of developmentally vulnerable children in the target suburbs of the Belconnen district in the ACT.

The following questions will be addressed:

1. Does a multi-sector, multi-component approach reduce the proportion of children who are considered developmentally at risk in the intervention community compared to the control community?
2. What is the reach, participation, intervention quality and dose of the interventions delivered and what were the barriers to, and facilitators of, implementation?

## Methods and analysis

### Design and intervention

This study will use a quasi-experimental design to determine the effectiveness of creating an integrated service network on reducing developmental vulnerability in young children. The formative components of the project commenced in November 2020 and the project will conclude in March 2024. The intervention will take place in the Belconnen district, chiefly within services located in the nine identified target intervention suburbs, while the Tuggeranong district will serve as the ‘control’ site. These two districts are well-matched on socio-demographic characteristics and on early child development outcomes from the most recent three censuses. Data will be collected from the AEDC for comparison, but no intervention will occur within this district other than those that are to be expected in a large urban area. The intervention will be conducted using a ‘staggered’ approach, starting with four suburbs (in two clusters) in the first year and ending with all nine suburbs (four clusters) receiving the intervention by the fourth year. An integrated multi-sector programme will be delivered to children and families in multiple settings in the intervention suburbs through a wide range of programmes and services that focus on the 5 AEDC domains of childhood development: physical health and wellbeing, social competence, emotional maturity, language and cognitive skills, and communication and general knowledge. The list of programs to be implemented are presented in Appendix 1 in S1 File. To help achieve key aims of this project, a family liaison coordinator will be employed. The role of this coordinator will be to work within the community to connect families to services and to facilitate the creation of referrals for children with additional needs. In addition, the family liaison coordinator will work with services to better connect them to other related services and to the wider community.

To achieve the second aim of this project, allied health programs will be embedded and delivered ‘in place’ into early childhood services within the intervention suburbs. Through a partnership with the University of Canberra, students from speech pathology, occupational therapy, health, nutrition, and education disciplines will work with a clinical educator to implement programs into early childhood services as a part of their work integrated learning. The programs will either function as professional learning for ECEC educators and playgroup facilitators or provide on-site clinical advice and support.

To supplement the aims and objectives of this project, other existing services and activities will be delivered to target suburbs within the community. Formal partnerships, marked by the creation of memoranda of understanding between organisations and the project, will be developed to ensure a clear understanding of the aims and scope of the partnership as well as to facilitate the creation or modification of services and programs to better address the needs of children and their families. This may include creating new programs, providing resources or funding to facilitate existing programs within intervention suburbs, or expanding current programs to reach more children and families. Within intervention suburbs, ‘loose parts play’ and ‘nature play’ as enabling environments will be held in suitable local settings e.g. parks, community halls, public spaces [16]. These place-based activities will be open to the public in non-stigmatising ways to engage local parents and caregivers who may not have been previously involved with other services due to lack of accessibility.

### Participants

The nine suburbs were selected using AEDC data from three censuses: 2012, 2015 and 2018. Suburbs were considered for the intervention if they had shown a worsening trend in vulnerability in at least three EDI domains and the ‘vulnerable in one or more domains’ category. These suburbs were then grouped into four ‘clusters’ based on geographical location, as shown in Appendix 2 in S1 File. This was done in order to target communities where we hypothesize that better integrated services will maximise the reach and number of quality early development opportunities for children.

The services invited to participate in the project were selected if they met the following criteria. First, the service must be located within one of the nine intervention suburbs or belong to one of the partner organisations and be located within the Belconnen district. Second, the service must target children aged birth to five years. The service must also pass a risk assessment and have educators who are willing and able to complete the programs and consent to data collection and a recorded follow-up interview.

ECEC educators, directors, and parents must be able to provide written consent before participating in any programs. Parents must also provide consent for their children if they are involved in any programs. Children will be asked to verbally consent to being involved in any activity outside of their regular ECEC schedule. An information sheet detailing the program and the role and expectations of the educator will be provided to them prior to commencing and must be signed and dated by both the educator and the PMT before being accepted into the project. The information sheet will also stipulate that participants are free to withdraw at any time and that all data pertaining to the participant, including interview recordings, will be removed from the study if requested.

### Data collection

Measures relating to both the overarching project aims and individual interventions will be collected pre- and post-intervention. Measures of service integration will be collected annually throughout the project for both control and intervention sites.

#### Developmental vulnerability

The primary aim, to reduce the proportion of children who are developmentally vulnerable, will be measured by comparing results from the AEDC. A baseline measurement was recorded using the 2018 AEDC data for each intervention suburb. The comparative data will be taken from the 2024 AEDC results. The two timepoints will be analysed to see if there has been a significant decrease in the proportion of children in the ‘developmentally vulnerable in one or more domains’ category, as well as across all five EDI domains. The 2021 census data will not be used in this study due to disruptions to service delivery in 2020. Additionally, the interventions will have been running for less than a year at the time of the data collection, and therefore not enough dose will have been given to result in any significant impact on AEDC scores.

*Outcome measures*. Every three years the Early Development Instrument of the AEDC measures the five domains of early development described above in Australian children who are commencing their first year of full-time school. Under each of these domains, children are classified into one of three categories: developmentally on track, developmentally at risk and developmentally vulnerable. Summary indicators are then calculated for each community based on the proportions of children in each of these three categories. The primary focus for this study will be to reduce the proportion of children in the intervention community who are classified as developmentally vulnerable on one or more domains. We will also perform separate tests for significant change in the percentage of children at risk in each of the five individual EDIs.

*Sample size estimates*. Our sample size calculation is based on the AEDC ‘critical difference estimates for communities’ [17]. We will focus the intervention on those suburbs (SA2) in the two poorest performing districts with the highest proportions of children with at least one EDI domain in the developmentally vulnerable category at the most recent census. For example, based on 2018 census results, ~40% of this sample have been classified as developmentally ‘at risk’, i.e. falling significantly behind their peers, and could benefit from early intervention. We anticipate that approximately half of this number, ~250–300 children, will be required to show a statistically significant difference. Our primary focus will be on reducing the number of children who are developmentally vulnerable on at least one index (Table 1). We will also perform separate tests for change in the percentage of children at risk in each of the five EDIs.

**Table 1:** Critical differences (%) to establish significant change for each of the six early development indices.

Community size (# of children)	Developmentally vulnerable (1+) (%)	Physical Health & wellbeing (%)	Social competence (%)	Emotional maturity (%)	Language & cognitive skills (%)	Communication & general knowledge (%)
200–299	4.7	5	4.6	4.9	4.8	5.2

Time-point comparisons will be made between 2021 and 2024 AEDC indices for both intervention and comparator areas. Across-area comparisons will be made between intervention and comparator areas for the 2024 census indices.

#### Service integration

Service integration will be measured mid-way through 2021 with respect to a 2019 baseline, and then annually for the duration of the project, using two validated tools. First, the Human Services Integration Measure (HSIM) by Browne et al. [18] will be given to all relevant organisations within the Belconnen district and all services and programs within the nine intervention suburbs. The tool (Appendix 3 in S1 File) is designed to gauge how each service perceives their current level of collaboration with other related services. To better reflect the context of the integration, the rating scale in the tool will be slightly adapted. Adaptations include a slight rewording of the rating explanations to better reflect the nature of service integration for early childhood centres and services and the addition of questions asking services to list the names of other services they have been involved with that did not appear on the survey. The tool will be distributed and completed using Qualtrics^XM^ (Qualtrics Australia, Sydney) online survey software. This will ensure the form is easy to complete and that all completed forms are returned and filed for data analysis.

The second tool, Successful Collaborations by Winkworth and White [19] will be administered to services that have indicated collaborations with other services on the HSIM tool. The Successful Collaborations tool (Appendix 4 in S1 File) aims to quantify the depth and scope of a collaboration between two services across three domains: capacity, authority, and shared value. The tool will be distributed, completed, and the data analysed online.

Representatives will also be invited to participate in a recorded interview. This interview will explore the collaboration in more depth and attempt to understand how service providers understand and use the term ‘integration’ and how they perceive the value and benefit of integrating services.

#### Within intervention measures

Within the allied health programs embedded into early childhood services and the publicly available programs, such as community playgroups and loose parts play sessions, measures of reach, dose, fidelity, environment, and outcomes will be gathered.

For the *Learning Language and Loving It* speech pathology program, several measures will be collected. The Teacher Interaction and Language Rating Scale (TILRS) [20] will be used to measure the effects of the intervention on the instructional practices and skills of the early childhood educators before and after participating in the program. Other child measures including phonological and print knowledge tests and receptive/expressive one-word picture vocabulary tests will be employed by the clinical educator and speech pathology students to gauge the effects of the intervention on children’s linguistic productivity and emergent literacy skills.

Measures for occupational therapy programs and support will include detailed field notes and program specific outcomes. The measures for the *Parents as Teachers* [21] child development program include process indicators such as the number of educators trained, and the number of sessions completed (taking in retention and staff churn). Facilitators will also collect data on the number of children from each intervention suburb that will be reached by the program.

Tools to assess the ECEC environment will also be used. The Early Language and Literacy Classroom Observation (ELLCO) [22] tool will be used to assess how well each centre supports and encourages positive literacy practices. The checklist includes questions on the book area, book selection, book use, writing materials, and writing around the room [23]. The Early Childhood Environment Rating Scale (ECERS-E) [22] will be used to evaluate the quality of the classroom environment. The questionnaire covers space and furnishings, personal care, language and literacy, learning activities, interaction, and program structure [24]. This measure will be used to assess the ECEC’s ability to implement the skills and practices they have learnt during the intervention and to foster and encourage early literacy and language skills.

Two measures will be collected from the student-led Kids at Play program (based on the Kids at Play Active Play program). A pre- and post-program survey will be administered to participants to gauge educator confidence to teach fundamental movement skills and promote active play and educators’ perceptions of the benefits of play. This survey was created by ACT Health but will have amendments from the Good Start In Life team. An Active Play Audit tool [25] will also be completed with the centre educators and/or director. This tool assesses the centre environment and aids educators in creating an active play policy and a Quality Improvement Plan for the centre, which will be re-evaluated 6 months post-intervention.

The community playgroups and loose parts play sessions will have sign-in sheets that will capture demographic information on the children attending. This includes age, cultural background, and gender of each child, and which suburb each family lives in (Appendix 5 in S1 File). No identifying information will be collected from any participant. Suburb information will be collected to assess the proportion of children attending from the intervention suburbs.

### Data analysis

#### Qualitative analyses

Interviews will be audio taped, transcribed verbatim and imported into NVIVO qualitative data analysis software. We will be following the procedure outlined by Braun et al. (2019) for using thematic analysis to analyse qualitative data [26]. Interview transcripts and observation narratives will be coded thematically by two researchers independently. Details will be collected on the contents of the interventions, service experiences of collaboration and the family liaison coordinator role.

#### Quantitative analysis

Testing for statistically significant differences in children’s early development indices will be based on the critical difference approach developed for the AEDC by Gregory and Brinkman [17]. The Gregory and Brinkman report provides the technical details to determine “how big” a difference (%) needs to be in the EDI results between two different time points or two different areas to be considered statistically significant [11, Tables 14 –primary outcome and 13 –the five secondary outcomes, respectively]. The method allows for the expected sample to sample variation and typical measurement error associated with the sort of data collected in the AEDC. Current versions of the methodology allow us to compare two point-estimates, either across time or between neighbourhoods, of the selected index. Table 1 shows the critical differences that will be needed to establish a significant change for each of the six EDI indices,—the primary outcome and five secondary outcomes—to be considered in this study. The population of children in the intervention and control areas is expected to be in the range 200–299 at the 2024 census. Time-point comparisons will be made between 2021 and 2024 AEDC indices for both intervention and comparator areas. Across-area comparisons will be made between intervention and comparator areas for the 2024 census indices. The following hypotheses will be tested for each of the six indices to be used in this study:

1. Each EDI for the intervention suburbs (within the Belconnen district) for the 2024 census will be significantly lower, i.e. show a significant reduction in the proportion of children who are developmentally vulnerable or at risk, compared to that in the control suburbs (within the Tuggeranong district)
2. Each EDI for the intervention suburbs (within the Belconnen district) for the 2024 census will be significantly lower, i.e. show a significant reduction in the proportion of children who are developmentally vulnerable or at risk, compared to the corresponding value in the 2021 census

## Discussion

In this paper, we present the design of a quasi-experimental study in which we conduct a process and outcomes evaluation of a multi-sector, multi-component programme of interventions for improving the physical and mental health of children who are at the highest risk of developmental vulnerability and disadvantage in the ACT. The focus of providing a family liaison coordinator in important behaviour settings within the child’s environment, i.e., early childhood education, playgroups and community settings, is considered a strength of this intervention.

The interventions will be implemented in collaboration with local professionals and community members. Community co-design during conceptualisation, development and planned implementation is central to this study. Participatory approaches ensure end-users and service provider voices are heard and provide an understanding of local practice and context to ensure more authentic engagement with community members in the target areas.

In addition to the outcome evaluation, a process evaluation will be conducted to provide insights into the implementation of the interventions and the role of the local context. The link between effectiveness and implementation are important for developing and scaling future health promotion interventions.

To monitor intervention effectiveness multiple data collection tools are used to measure the different outcomes. Firstly, fidelity measures are collected to ensure the programs and interventions are implemented as intended. Incorrectly or incompletely implemented programs may result in the intervention having no effect on educator professional knowledge and practice, and therefore these measures must be collected to account for any lack of significant differences in the primary outcome measure. These outcomes will be detailed in the process evaluation. Other data collected, including the AEDC results, service integration survey scores, and data from the speech pathology and occupational therapy programs, will be used to form the outcome evaluation. This evaluation is used to assess the extent to which the project delivered the outcomes outlined in the planning program logic and if there were any significant improvements in developmental vulnerability in children in the Belconnen district that may have been influenced by this project.

This study has several limitations. The primary limitation arises from the use of the AEDC data. For confidentiality and privacy, the AEDC only releases suburb level data. However, it is widely understood that children do not always attend ECECs and services within the suburb in which they live. As a result, providing place-based interventions within intervention suburbs may not reach as many children from that suburb as intended. The study will collect data on which suburb each child attending a program or service lives in, but purposely excluding children from other suburbs is not possible as it is unethical and defies the universal nature of the programs and services provided.

It is known to the PMT that another similar project is operating in the Belconnen District at the same time as the Good Start in Life project. Whilst efforts have been made to regularly communicate and collaborate with the other project, there is the chance that their work may interfere with the two primary outcome measures of this project. Additionally, early childhood education centres, services and programs may elect not to engage with the study and to conduct their own professional development practices or engage with other services on their own. This may also influence the data this project is collecting on child development outcomes and service integration.

Finally, there is the chance that similar projects and interventions may operate within the control site, the Tuggeranong District. Once again, it is unethical and unfeasible to prevent other research teams or organisations from implementing similar programs in that area. However, throughout the study we will record any new programmes/services that are delivered in this control area.

### Patient and public involvement statement

Extensive consultation with key services and local stakeholder organisations/groups have been undertaken to inform study design and programme implementation. An advisory board has been established with representation from key partners (and from health, social care and education sectors) which meets every three months.

## Supporting information

S1 File Contains all supporting Appendices 1–5. (DOCX)
