# Supplementary material for: A Good start in life: Effectiveness of integrated multicomponent multisector support on early child development—Study protocol
Source: PLoS One. 2022 Aug 3;17(8):e0267666. doi: 10.1371/journal.pone.0267666 (PMC9348669; doi:10.1371/journal.pone.0267666)
Supplement: S1 File — (DOCX) [file pone.0267666.s001.docx]

# Appendix 1 – List of Services for young children in the Belconnen District

| **SECTOR** | **NAME of PROGRAM** |
| --- | --- |
| Education | For profit and NGO ECEC’s  ACT Playgroups – community and early intervention/supported.  Preschool – ACT Education Directorate |
| Health | **WBCFC**  *ACT HEALTH*  Child Health Clinic & drop in clinic  Early days  Immunisation  Sleep and settle  Fussy eaters  Physio  Nutrition  ***CSD***  Child development service (Speech & Physio)  **Belconnen Community Health Centre**  MACH Health Checks  Community Paediatric and Child Health Services  Asthma education  Eye screening  Fussy Eaters  Child at Risk Health Unit |
| Community/social support | **ACT Playgroups**  Early intervention playgroup  **WBCFC**  Get Up (parents under 25 yrs)  Koori playgroup  Learn giggle and grow  Parents as Teachers  PoPPY (parents with MH)  South Sudanese group mandarin for fun  Children’s Behaviour and Emotional Wellbeing Clinic  Circle of Security relationship-based parenting program  Cool Little Kids  Tuning into Kids  Drop in Services:  New parent groups  DVCS Outreach  MARRS Outreach  Marymead Kayak program (post separation)  YWCA circle of support  **CRCS**  Belconnen ECEC  Ginninderra ECEC  Bruce ECEC  Calvary ECEC  Family Foundations  Circle of Security  Paint and Play  Bringing up Great Kids  Creative Arts Group  Early English  Early Links to Learning  Fit Families  Multicultural playgroup  Tiny Tumblers  Tuning in to Kids |
| Disability | EACH Programme |

# Appendix 2 – Map of intervention and control clusters


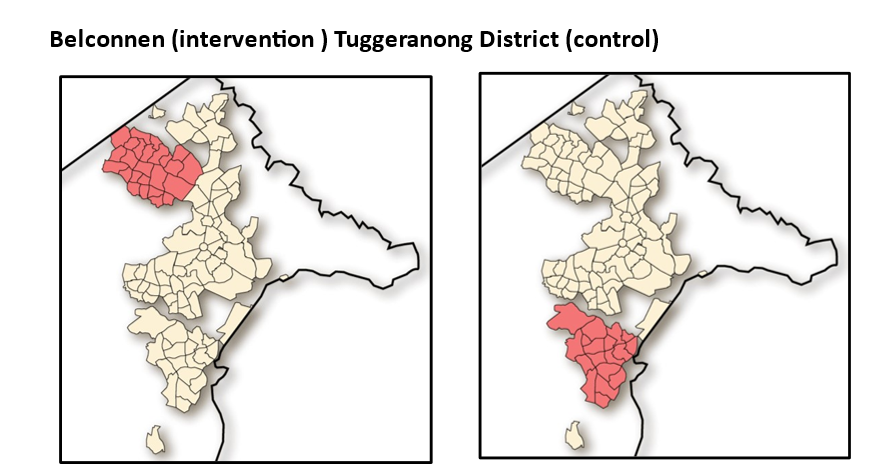


# Appendix 3 – The Human Services Integration Measure


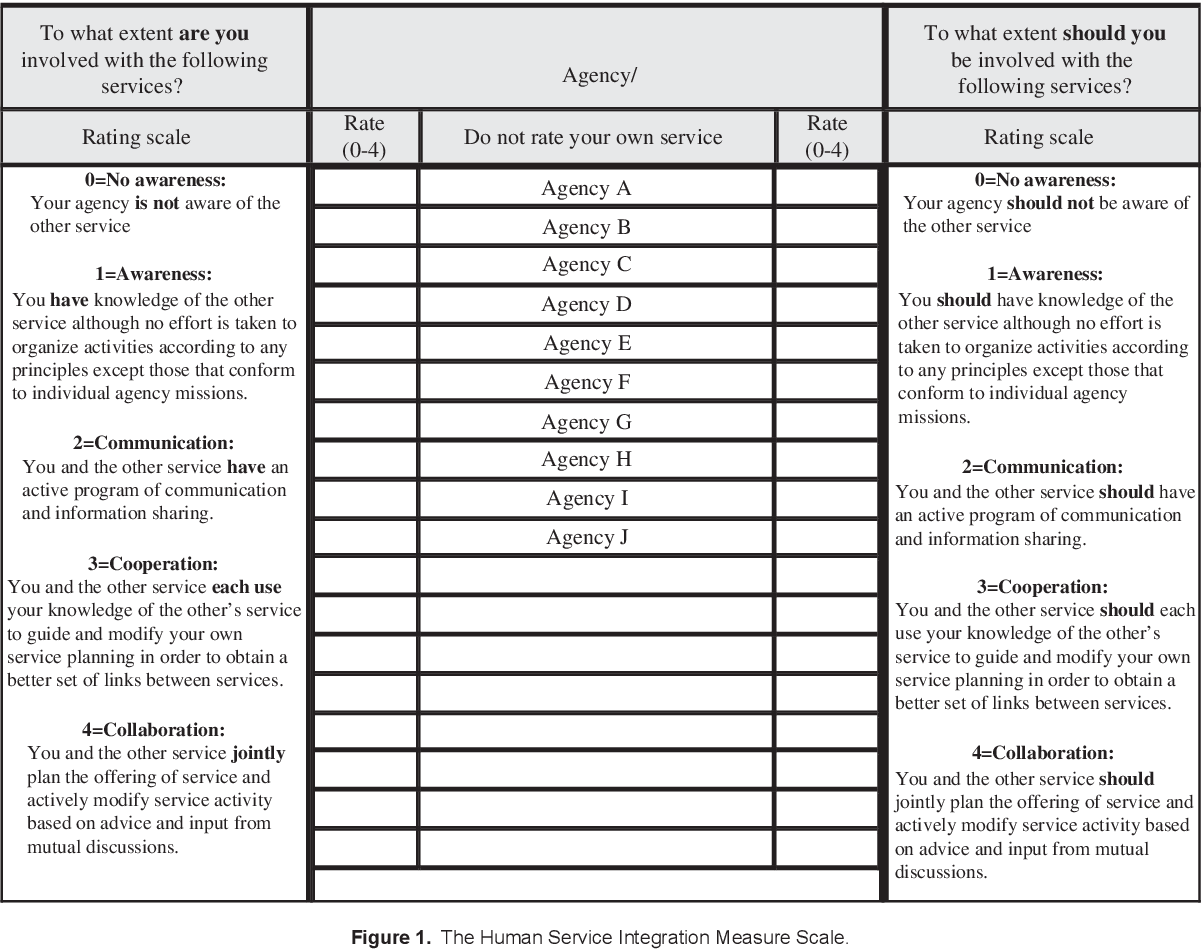


#
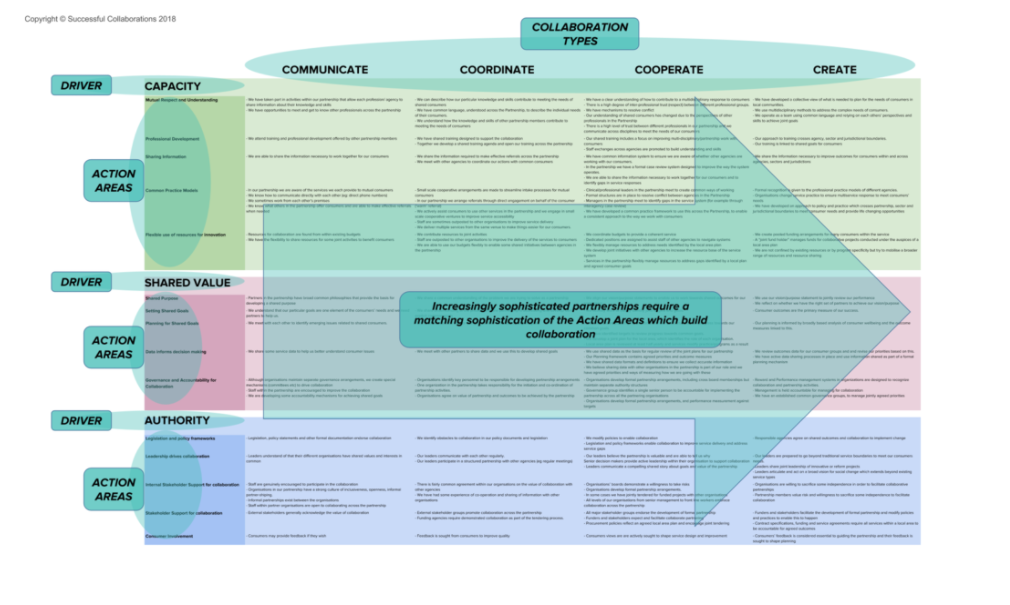
Appendix 4 – The Successful Collaborations Tool Rubric

# Appendix 5 - Loose Parts Play Sign In Sheet

| **Age of Child** | **Gender** | **Cultural Background** | **Suburb of Residence** |
| --- | --- | --- | --- |
|  |  |  |  |
|  |  |  |  |
|  |  |  |  |
|  |  |  |  |
|  |  |  |  |
|  |  |  |  |
|  |  |  |  |
|  |  |  |  |
|  |  |  |  |
|  |  |  |  |
|  |  |  |  |
|  |  |  |  |
